# It is time to bring borderline intellectual functioning back into the main fold of classification systems

**DOI:** 10.1192/pb.bp.115.051490

**Published:** 2016-08

**Authors:** Jannelien Wieland, Frans G. Zitman

**Affiliations:** 1 Kristal Centre for Psychiatry and Intellectual Disability, Rivierduinen, The Netherlands; 2 Leiden University Medical Centre, The Netherlands

## Abstract

Borderline intellectual functioning is an important and frequently unrecognised comorbid condition relevant to the diagnosis and treatment of any and all psychiatric disorders. In the DSM-IV-TR, it is defined by IQ in the 71–84 range. In DSM-5, IQ boundaries are no longer part of the classification, leaving the concept without a clear definition. This modification is one of the least highlighted changes in DSM-5. In this article we describe the history of the classification of borderline intellectual functioning. We provide information about it and on the importance of placing it in the right context and in the right place in future DSM editions and other classification systems such as the International Classification of Diseases.

One of the least highlighted changes in the DSM-5 is the modification of the classification of borderline intellectual functioning.^1^ Contrary to earlier DSM versions, IQ boundaries are no longer part of the classification, leaving the concept without a clear definition. IQ scores are, over most of the range, well described by a normal distribution. The term borderline intellectual functioning describes a group of people who function on the border between normal intellectual functioning and intellectual disability, between 1 and 2 standard deviations below the mean on the normal curve of the distribution of intelligence, roughly an IQ between 70 and 85. According to the normal curve, as much as 13.6% of the population falls into this category.

Borderline intellectual functioning has always been a difficult concept. It had different names, different boundaries, and travelled through earlier DSM editions starting as a solid element of mental deficiency in the DSM-I and ending, in DSM-5, as a V-code literally in the last place. Its classification in DSM-5 has followed a similar path as in the ICD.^2–4^ And although ICD-11 is not due until 2017, it will likely share the same view as DSM-5.

In DSM-5, similar to the new classification of intellectual disability, IQ test scores are removed from the diagnostic description of borderline intellectual functioning. This is detrimental to the concept, since it was the only criterion left. Within the classification of intellectual disability, even in DSM-5, the importance of standardised IQ scores is well described. As a V-code, borderline intellectual functioning ultimately has been defined solely by IQ, but by removing the IQ criterion, DSM-5 no longer provides any criteria for what exactly borderline intellectual functioning is. ICD-11 will probably do the same or might score out the classification of borderline intellectual functioning altogether. This is in spite of the fact it is critically an important and frequently unrecognised comorbid condition vastly relevant to the diagnosis and treatment of any and all psychiatric disorders. Here we describe the history of the classification of borderline intellectual functioning and provide information about it as well as on the importance of placing it in the right context and in the right place in future editions of the DSM and other classification systems such as the ICD.

## History of borderline intellectual functioning

### DSM

Before DSM-I, different cut-offs in IQ scores were used when it comes to what is now called intellectual disability or intellectual developmental disorder. Consequently, intellectual disability included what we now call borderline intellectual functioning. In DSM-I, borderline intellectual functioning was called mild mental deficiency, listed in the section ‘Mental Deficiency’.^5^ The classification applied when there was both an IQ of about 1 to 2 standard deviations below the mean (equalling an IQ between 70 and 85) and functional impairment. DSM-I already made a plea for a classification based on more than a standardised IQ test alone. It states that cultural, physical and emotional determinants, as well as school, vocational and social effectiveness, should be taken into consideration.^5^

In DSM-II, borderline intellectual functioning was called borderline mental retardation. It had a place in the section ‘Mental Retardation’.^6^ The boundaries of borderline mental retardation (IQ 68–83) differed slightly from those of mild mental deficiency in the DSM-I, but the other criteria remained the same. The place of borderline intellectual functioning dramatically changes in DSM-III,^7^ where it is no longer part of what were then called intellectual disorders. Mental retardation is now covered in the chapter of disorders usually first evident in infancy, childhood or adolescence, whereas borderline intellectual functioning is now a V-code ‘exiled’ to the chapter ‘V-Codes for Conditions Not Attributable to a Mental Disorder that are the Focus of Attention or Treatment’ in the far back of the DSM. The V-code borderline intellectual functioning is to be used when the focus of attention or treatment is associated with borderline intellectual functioning, i.e. an IQ in the 71–84 range. Where in the classification of mental retardation it is still recognised and described that IQ should not be the only criterion in making a diagnosis of mental retardation or in evaluating its severity, in the V-code borderline intellectual functioning the IQ is now the only criterion left.

### ICD

In the ICD, borderline intellectual functioning has a similar history, being excluded from the section of mental retardation at about the same time as in the DSM.^2,3^ This change obviously caused a significant decrease in the prevalence of intellectual disability. In the DSM-III the argument was that the large majority of individuals with borderline intellectual functioning does not have significantly impaired adaptive behaviour.^7^ For more than 30 years the classification of borderline intellectual functioning then does not change and the DSM-III, DSM-IV and DSM-IV-TR all use the V-code it was assigned. During the same time in the ICD borderline intellectual functioning got shifted to the residual code R41.8, a rather non-specific code referring to ‘other and unspecified symptoms and signs involving cognitive functions and awareness’.^4^

Now, DSM-5 has further stripped the definition of borderline intellectual functioning. The V-code is listed in ‘Other Conditions That May Be a Focus of Clinical Attention’, under ‘Other Circumstances of Personal History’. It ceases to provide any description of what borderline intellectual functioning entails. The DSM-5 just states that the V-code can be used when an individual's borderline intellectual functioning is the focus of clinical attention or has an impact on their treatment or prognosis.

## Vulnerability and mental health

According to DSM and ICD, borderline intellectual functioning is not a disorder. But people with borderline intellectual functioning, or an IQ between 70 and 85, do comprise a vulnerable group. Genetic liability, biological causes such as perinatal difficulties, and epigenetic factors such as socioeconomic status and maternal stress all contribute to borderline intellectual functioning.^8^ Children with borderline intellectual functioning are uniquely at risk for receiving poor parenting.^9^ Mothers of children with borderline intellectual functioning were less positive and sensitive, and showed less positive engagement, even though their children did not exhibit more difficult child behaviour. Given the importance of positive and sensitive parenting for secure attachment and adaptive regulatory capabilities, children with borderline intellectual functioning might be at risk at a very early age.^9^ In adult life, contrary to the DSM-III statement, perhaps increasingly so owing to the growing complexity of society, many people with borderline intellectual functioning do have problems in adaptive functioning. In fact, they face difficulties across all areas of ordinary life.^10–14^ They are at increased risk of experiencing physical problems,^13^ poverty,^15^ have more difficulties with activities of daily living,^11^ have limited social support^11,12^ and no access to specialised services.^15^ They often live problematic lives, functioning under high strain but unnoticed by the rest of society.^15^ Many people with borderline intellectual functioning do not have psychiatric disorders, but they are more vulnerable to the development of mental health problems than people of average or above average intelligence and may also be more vulnerable than people with mild intellectual disability.^11,15–20^ Several studies show increased risk for the development of almost all psychiatric disorders in childhood as well as in adulthood, including substance misuse and personality disorders.^11,12,16,17,21,22^

When such people do develop psychiatric disorders, especially when there are additional problems in adaptive functioning, borderline intellectual functioning and its impact on the presentation, diagnostics and treatment of the psychiatric disorder should not be overlooked. Even though borderline intellectual functioning is not a disorder, nor a disability in itself, it is an impediment in diagnostics and treatment when a psychiatric disorder develops.

Unfortunately, despite the fact that borderline intellectual functioning is a vulnerability and has impact on comorbid disorders, people who are thus affected are almost invisible in research and – when they develop comorbid psychiatric disorders – are rarely identified as having borderline intellectual functioning in mental healthcare. The DSM-5 classification as is, is not likely to improve on this problem.

## Why paying serious attention to borderline intellectual functioning remains important

As is the case with many psychiatric diagnoses, being classified as having borderline intellectual functioning can have a stigmatising effect. People with a low IQ often try to prevent their limited intellectual capabilities being exposed by painstakingly trying to behave ‘normally’ and masking their disabilities and special needs. Also, society as a whole tries to look away from borderline intellectual functioning. For example, people thus affected are not entitled to the special support services for people with intellectual disability because their IQ is deemed too high. Unlike other countries, in The Netherlands, individuals with borderline intellectual functioning and comorbid psychiatric disorders are eligible to the same specialised mental healthcare services as people with intellectual disability. In this way special attention to the impact of borderline intellectual functioning on comorbid psychiatric disorders and the special skills training that mental health workers need to treat this patient group adequately are guaranteed. It is doubtful whether general mental healthcare services are able to deliver the same adequate care. It is important to take borderline intellectual functioning into account as a complicating factor from the start of diagnostics and treatment and to train mental health workers in this respect.

At this time, however, most mental healthcare professionals are not trained in recognising borderline intellectual functioning and miss the extra skills needed for effectively treating psychiatric disorders in such patients. Psychiatric patients with borderline intellectual functioning in regular mental healthcare are more likely to get psychotropic drugs (and more likely antipsychotics and sedatives than antidepressants) than psychotherapy.^11,23^ Psychiatric treatments in regular mental healthcare are not adapted to the cognitive limitations of such patients, such as deficits in memory function.^17^ Results from daily clinical practice show that many forms of treatment can be adapted to the cognitive abilities of such people but also that neglect of borderline intellectual functioning in psychiatric patients leads to longer duration of treatment, more need for crisis intervention^11,18^ and limited or even adverse treatment effect.

## Conclusions

We recommend a renewed regard to the concept of borderline intellectual functioning and its place in the DSM and other classification systems such as the ICD. A well-defined classification can improve visibility of patients with borderline intellectual functioning in mental healthcare, bridging the gap between high prevalence and low recognition. Recognition and acknowledgement of patients with borderline intellectual functioning and attention to their specific mental healthcare needs is likely to substantially improve the quality of mental healthcare for a group of people that comes last too often.
